# A *Rab33b* missense mouse model for Smith-McCort dysplasia shows bone resorption defects and altered protein glycosylation

**DOI:** 10.3389/fgene.2023.1204296

**Published:** 2023-06-08

**Authors:** Milena Dimori, Irina D. Pokrovskaya, Shijie Liu, John T. Sherrill, Horacio Gomez-Acevedo, Qiang Fu, Brian Storrie, Vladimir V. Lupashin, Roy Morello

**Affiliations:** ^1^ Department of Physiology and Cell Biology, University of Arkansas for Medical Sciences, Little Rock, AR, United States; ^2^ Department of Orthopaedic Surgery, University of Arkansas for Medical Sciences, Little Rock, AR, United States; ^3^ Department of Biomedical Informatics, University of Arkansas for Medical Sciences, Little Rock, AR, United States; ^4^ Department of Internal Medicine, Division of Endocrinology, University of Arkansas for Medical Sciences, Little Rock, AR, United States; ^5^ Division of Genetics, University of Arkansas for Medical Sciences, Little Rock, AR, United States

**Keywords:** Smith-McCort dysplasia, RAB33B, GTPases, bone, Golgi, glycosylation

## Abstract

Smith McCort (SMC) dysplasia is a rare, autosomal recessive, osteochondrodysplasia that can be caused by pathogenic variants in either *RAB33B* or *DYM* genes. These genes codes for proteins that are located at the Golgi apparatus and have a role in intracellular vesicle trafficking. We generated mice that carry a *Rab33b* disease-causing variant, c.136A>C (p.Lys46Gln), which is identical to that of members from a consanguineous family diagnosed with SMC. In male mice at 4 months of age, the *Rab33b* variant caused a mild increase in trabecular bone thickness in the spine and femur and in femoral mid-shaft cortical thickness with a concomitant reduction of the femoral medullary area, suggesting a bone resorption defect. In spite of the increase in trabecular and cortical thickness, bone histomorphometry showed a 4-fold increase in osteoclast parameters in homozygous *Rab33b* mice suggesting a putative impairment in osteoclast function, while dynamic parameters of bone formation were similar in mutant *versus* control mice. Femur biomechanical tests showed an increased in yield load and a progressive elevation, from WT to heterozygote to homozygous mutants, of bone intrinsic properties. These findings suggest an overall impact on bone material properties which may be caused by disturbed protein glycosylation in cells contributing to skeletal formation, supported by the altered and variable pattern of lectin staining in murine and human tissue cultured cells and in liver and bone murine tissues. The mouse model only reproduced some of the features of the human disease and was sex-specific, manifesting in male but not female mice. Our data reveal a potential novel role of RAB33B in osteoclast function and protein glycosylation and their dysregulation in SMC and lay the foundation for future studies.

## Introduction

Smith-McCort (SMC) dysplasia and Dyggve-Melchior-Clausen (DMC) dysplasia are similar, rare, autosomal recessive, osteochondrodysplasias that share identical radiologic features and cartilage histology (Kaufman et al., 1971; Beighton, 1990; Nakamura et al., 1997; Neumann et al., 2006). Skeletal defects present after birth, usually between 18 and 48 months of age, and are progressive resulting in significant deformities (Tuysuz et al., 2021). Typical features of the disease are short trunk dwarfism with barrel-shaped chest, short neck, double-humped vertebral bodies, platyspondyly, and lacy iliac crests, which can be accompanied by additional defects such as genu valgum, brachydactyly, joint contractures and others (Tuysuz et al., 2021). DMC patients can be distinguished from SMC patients by the presence of intellectual disability and coarse facies (Nakamura et al., 1997; Burns et al., 2003; Dupuis et al., 2015). The first genetic locus for Smith-McCort dysplasia (SMC1) was identified on chromosome 18q21 and associated with homozygous or compound heterozygous pathogenic variants in the *DYM* gene (encoding Dymeclin) (Cohn et al., 2003). Pathogenic variants in the same gene also cause Dyggve-Melchior-Clausen disease, making SMC and DMC allelic disorders (Ehtesham et al., 2002; El Ghouzzi et al., 2003). Dymeclin is a poorly characterized intracellular protein involved in Golgi organization, intracellular vesicle trafficking, and the accumulation of extracellular cell surface collagens (Osipovich et al., 2008; Denais et al., 2011; Dupuis et al., 2015). A second locus for Smith-McCort dysplasia (SMC2) was identified on chromosome 4q31 (Alshammari et al., 2012; Dupuis et al., 2013). SMC2 is caused by homozygous or compound heterozygous pathogenic variants in the *RAB33B* gene, which encodes a small GTP-binding protein within the large superfamily of small GTPases. These enzymes function as molecular switches and regulate a variety of cellular processes by transducing intracellular information and alternating between an active GTP-bound and an inactive GDP-bound state (Cherfils and Zeghouf, 2013). RAB proteins perform their regulatory function by recruiting a variety of effectors to mediate different functions in membrane transport, including vesicle trafficking, docking and fusion (Hutagalung and Novick, 2011; Morgan et al., 2019; Roy and Roux, 2020). RAB33B localizes to the medial Golgi apparatus (Zheng et al., 1998; Starr et al., 2010; Pusapati et al., 2012; Morgan et al., 2019), and its depletion using siRNA resulted in a significantly increased number of Golgi-associated vesicles per stack, suggesting a functional role of the protein in vesicle trafficking at the Golgi apparatus level (Starr et al., 2010). RAB33B is also involved in membrane fusion events, e.g., between autophagosomes and lysosomes (Itoh et al., 2008) and in post-Golgi vesicular trafficking to the plasma membrane, and in particular in delivering β1 integrin cargo for the formation of focal cell contacts with the extracellular matrix (Bjornestad et al., 2022). A close paralog of *RAB33B* is *RAB33A*; while these two genes appear to co-regulate aspects of CNS development, *RAB33A* expression seems to be primarily restricted to the CNS while *RAB33B* is expressed in several tissues (Cheng et al., 2006; Huang et al., 2019). Thus, disease-causing variants in either *RAB33B* or *DYM* appear to affect the Golgi apparatus, the central organelle that coordinates protein processing, glycosylation and secretion. Because of the importance of the extracellular matrix (ECM) and of secreted growth/signaling factors during skeletal formation, development and homeostasis, we hypothesized that the underlying disease mechanism in SMC may be associated with defective Golgi-dependent protein processing/glycosylation leading to skeletal defects. However, the molecular connection between *RAB33B* genetic alterations and defects in skeletal development is unknown. We generated mice that carry a *Rab33b* missense disease-causing variant identical to that described in a consanguineous family where multiple members were diagnosed with SMC (Alshammari et al., 2012). To characterize the phenotype of this new mouse model, we performed a variety of *ex-vivo* and *in vitro* assays, including dual X-ray absorptiometry (DEXA) and micro-computed tomography (µCT) at 6 weeks (young) and 4 months (mature) of age in male and female mice, in addition to bone histology/histomorphometry, X-ray imaging, biomechanical tests, and lectin staining of cell and tissue sections. Our results indicate a mild phenotype consistent with osteoclast defective bone resorption and altered protein glycosylation with an impact on bone material properties.

## Methods

### Mouse generation, genotyping and ethic statement

**FIGURE 1:**
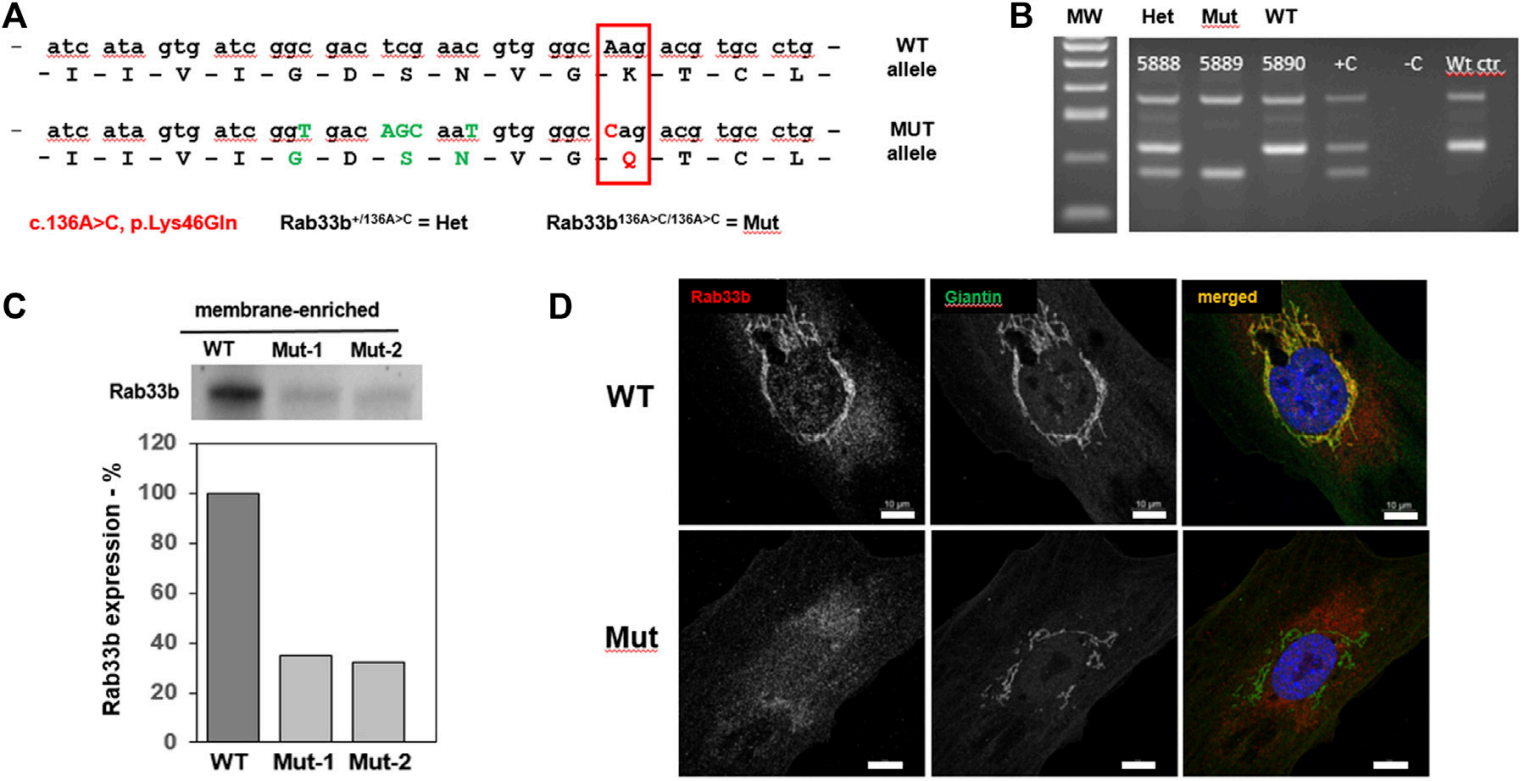
Generation of a new mouse model for SMC. **(A)**. Diagram of the partial nucleotide and amino acidic sequence of RAB33B indicating the c.136A>C (p.Lys46Gln) disease-causing variant (red box) introduced in the mutant allele as well as the upstream silent nucleotide changes shown in green fonts. Henceforth, mice carrying one mutant allele will be referred to as Het while homozygous mice will be referred to Mut. **(B)**. Example of a genotyping gel, including positive and negative control samples. **(C)**. Western Blot using a RAB33B antibody on membrane-enriched lysates of freshly dissected adult liver from 1 wild-type (WT) and 2 *Rab33b* mutant mice, separated by SDS-PAGE. Relative quantification of the bands is shown below the stained membrane. **(D)**. Immunofluorescence staining of RAB33B (red) and the Golgi marker Giantin (green) on primary calvarial osteoblasts. Scale bars = 10 μm.

The University of Arkansas for Medical Sciences (UAMS) IACUC committee approved all animal procedures performed in this study, which were conducted in accordance with local, State and US Federal regulations. Mice were housed in ventilated cages in a pathogen free facility at 22°C, in a 12-h light/dark cycle, and supplied with water and standard chow at libitum. The Rab33b p.Lys46Gln (NM_031296:c.136A>C) missense pathogenic variant was knocked into the mouse genome using a CRISPR/Cas9 approach by the local UAMS Transgenic Core facility; a few silent nucleotide changes upstream of the mutation were also introduced to facilitate mouse genotyping (see Figure 1). Four founder mice were obtained and bred with a C57B6 mate purchased from the JAX labs. Offspring were genotyped by PCR and the region of interest was sequenced (Sanger). Two males and two females (F1) were confirmed to be heterozygous for the desired mutation and were bred to generate heterozygous, homozygous mutant (*Rab33b* ^*A136C/A136C*^) and wild-type mice for the study. PCR genotyping was performed with the GoTaq G2 Hot Start Polymerase reagent (cat# M7423 Promega) using a Master Cycler thermocycler (Eppendorf). An example gel image of the genotyping results is shown in Figure 1B. For primer sequences and PCR conditions, please see Supplementary Material S1. The skeletal phenotype of male and female mice was analyzed at 6 weeks and at 4 months of age. Mouse body weights were obtained at each time points before tissue harvest.

### Cell culture and RNA interference

Primary calvarial osteoblasts were obtained from 3–5 days old pups as previously described (Morello et al., 2006) and were grown in Alpha-MEM, supplemented with 10% Fetal Bovine Serum (FBS), L-Glutamine (2 mM), 100 unit/ml penicillin, and 100 mg/ml streptomycin. Briefly, calvariae were dissected, washed in sterile PBS 3 times and digested with Collagenase P (1 mg/ml) in a 6-well plate for 20 min at 37°C with vigorous shaking every 5 min. After 20 min the medium was removed, and the digestion step was repeated. Then 1 ml of digestion media with double amount of collagenase was added and each calvaria was finely minced with sterile scissors, incubated for 1 h at 37°C, with vigorous shaking every 5–10 min. Alpha-MEM with 15% FBS complete medium (3.75 ml) was then added until the next day. Cells were then re-suspended with a pipette and allowed to attach to the plate for 4–6 h. Finally, the wells were washed twice with sterile PBS, 2 ml of culture medium was added to each well and the cells were grown for 24–48 h. HeLa cells stably expressing Golgi enzyme GalNAcT2-GFP were cultured in DMEM supplemented with 10% fetal bovine serum (FBS) and 0.45 mg/ml of Geneticin in a humidified incubator at 37°C and 5% CO2. siRNA directed against Rab33b has been described previously (Starr et al., 2010). Control siRNA was siControl, non-targeting siRNA (UAG​CGA​CUA​AAC​ACA​UCA​A). All siRNAs were manufactured by Dharmacon RNA Technologies. siRNA duplexes were transfected at a final concentration of 200 nM using Oligofectamine (Invitrogen) according to the manufacturer’s protocol with minor modifications. In brief, −70,000 HeLa cells stably expressing GalNAcT2-GFP were seeded per 35 mm tissue culture dish containing 12 mm diameter coverslips (Fisher Scientific). Cells were cultured overnight and then transfected with the corresponding siRNA in the absence of FBS. To achieve maximal knockdown, second cycle of siRNA transfection was performed 24 h after the initial transfection. 96 h after the first transfection cycle, cells were fixed with 1% paraformaldehyde for immunofluorescence staining (300 cells counted and mean pixel intensity averaged) or lysed for Western blotting to determine Rab33B protein knockdown level. For the osteoclastogenesis assay, please see Supplementary Material S1

### Western blot, immunofluorescence, and lectin staining

HeLa cells were lysed in 2% SDS, followed by standard SDS–PAGE (−12% acrylamide) and Western blotting. Antibodies used for Western blotting (WB) were anti-Rab33B (Frontier Institute, Clone D5) and anti-α-tubulin (Sigma-Aldrich). The blots were scanned and analyzed with an Odyssey Infrared Imaging System (LICOR Biosciences). HeLa cells stably expressing GalNAcT2-GFP were transfected with corresponding siRNA as described above, and then fixed with 1% paraformaldehyde and blocked with 0.1% BSA in PBS. Cells were incubated for 30 min with Alexa Fluor 555 conjugated WGA lectin or Alexa Fluor 488 conjugated GSII lectin diluted in PBS. Confocal image stacks were taken for the analysis of Rab33B and GalNAcT2-GFP distribution, while wide-field images were captured for surface lectin distribution. Both wide-field images and confocal image stacks were collected with a 63x/1.40 numerical aperture objective and a Zeiss 200M inverted microscope. Confocal image stacks were produced with a BD CARV II spinning disk confocal accessory mounted on the microscope. Images were processed with iVision-MAC software.

Preparation of mouse tissue and cell lysates and WB analysis was done as described earlier (Sumya et al., 2021). Please also see Supplementary Material S1. For WB analysis 10–20 µg of protein was loaded into Genescript (8%–16%) gradient gel. Proteins were transferred onto nitrocellulose membrane using the Thermo Scientific Pierce G2 Fast Blotter. Membranes were washed in PBS, blocked in Odyssey blocking buffer (LI-COR) for 20 min, and incubated with primary antibodies rRab33 (Santa Cruz, 1:500) or lectin Helix Pomatia Agglutinin (HPA)-Alexa 647 (Thermo Fisher, 1:1000) for 1 h at room temperature or overnight at 4°C. Membranes were washed with PBS and incubated with secondary fluorescently-tagged antibodies (Alexa Fluor 647 Donkey anti-mouse, Jackson Immuno Research/705605–151, 1:8000) diluted in Odyssey blocking buffer for 1 h. Blots were then washed with PBS and imaged using the Odyssey Imaging System. Images were processed using the LI-COR Image Studio software.

### Super-resolution AiryScan fluorescent microscopy

Immunofluorescence microscopy was done using the previous protocol (Willett et al., 2013) with some additional modifications. Briefly, primary mouse osteoblasts grown on 12-mm round coverslips to 80%–90% confluence were fixed with paraformaldehyde (PFA, freshly made from 16% stock solution) diluted in phosphate-buffered saline (PBS) for 15 min at room temperature. For the lectin staining, 1% PFA was used for fixation, followed by incubations with 50 mM ammonium chloride for 5 min and two incubations in the blocking buffer (0.1% BSA in PBS). After that, cells were incubated with HPA-647 diluted in blocking buffer for 30 min. For the antibody staining cells were fixed with 4% PFA, treated with 50 mM ammonium chloride (5 min), and permeabilized with 0.1% Triton X-100 (1 min) followed by two incubations with the blocking buffer. After 45 min incubation with primary antibodies: Giantin (Covance PRB-114C rabbit 1:100 and rRab33b (Frontier Institute Clone D5, mouse 1:30, diluted in the antibody buffer (1% cold fish gelatin, 0.1% saponin in PBS), cells were washed three times in PBS and incubated with fluorescently conjugated secondary antibodies diluted in antibody buffer for 30 min. Cells were washed four times with PBS, then coverslips were dipped in PBS and water 10 times each and mounted on glass microscope slides using Prolong^®^ Gold antifade reagent (Life Technologies). Cells were imaged with a 63 × oil 1.4 numerical aperture (NA) objective of an LSM880 Zeiss Laser inverted microscope with Airyscan using ZEN software. Labeling of unmasked paraffin sections with fluorescently labeled lectins (HPA-647, 1:500, Wheat Germ Agglutinin-Rhodamine Red, 1:500, GNL-Alexa 647 1:500) was performed as above.

### Dual-energy X-ray absorptiometry and digital X-ray imaging

A DEXA scanner (PIXIMUS2, Lunar, Madison, WI) was used after sacrifice to determine the femur, lumbar spine, and whole body bone mineral content (BMC) and bone mineral density (BMD) in 6 weeks and 4 months old mice, according to standard procedures. One full scan per mouse was performed and analyzed with PIXImus software 2.1 (GE/Lunar). The head and the neck were excluded from whole body calculations by drawing a ROI. The PIXImus was calibrated with a phantom (corresponding to bone mineral density = 0.0622 g/cm2 and 11.3% fat) on each day of testing according to the manufacturer’s instructions.

The skeleton of a select group of mice, including both male and females mice at different age, was also digitally X-ray imaged using an UltraFocus Faxitron instrument (Hologic).

### Micro-computed tomography

Femurs and lumbar spines were dissected from male and female mice at 6 weeks and 4 months of age and fixed in 95% ethanol (excluding the 4 months old femurs that were frozen instead for biomechanical assessment). Femur lengths were measured using a digital caliper. Micro-CT analysis was performed on a MicroCT 40 (Scanco Medical AG, Bassersdorf, Switzerland) using a 12 μm isotropic voxel size. For details, please see Supplementary Material S1 Bone trabecular and cortical parameters were determined as described (Suva et al., 2008). Standard nomenclature guidelines were followed to report all micro-CT measurements (Bouxsein et al., 2010).

### Biomechanics

Femurs from 4 months old male mice were harvested, wrapped in saline-soaked gauze and frozen at −20. Bones were allowed to thaw for at least 2 h at room temperature before micro-CT scanning. After micro-CT scanning, these same femurs were tested in a 3-point bending test using an ElectroForce 5500 Test Instrument (TA Instruments, Delaware, United Stataes) with a ramp rate of 0.05 mm/s and support span of 8 mm, and running WinTest software version 8.2. Moment of Inertia (MOI) values were derived from micro-CT scans of the same femur. Load-displacement curves were generated for each sample and representative curves are shown in Figure 7B. Structural properties of stiffness, yield and maximum load, post-yield displacement and work-to-fracture were calculated for each sample. Material properties of Young’s Modulus, yield and maximum stress, were also calculated for each sample.

### Histology and histomorphometry

For the assessment of the epiphyseal growth plate and measurement of the bone formation rate, mice were injected by i.p. with 30 mg/kg Alizarin red (alizarin-3-methyliminodiacetic acid, Sigma cat: A3882) and calcein (Sigma cat: C0875) dyes at 6 and 2 days before sacrifice, for the 6 weeks old mice, and at 7 and 2 days before sacrifice for the 4 months old mice. Right legs and lumbar spines were collected, fixed in Millonig’s solution overnight, and dehydrated in graded series of increasingly concentrated ethanol before embedding in methyl-methacrylate according to standard procedure (Gruenwald et al., 2014). Serial, coronal, 5 µm thick sections through the center of the vertebral body of L3 and L4 were generated and the entire cancellous bone area of L4 was analyzed. Sections were stained with Tartrate-resistant acid phosphatase (TRAP) and counterstained with Fast Green for counting osteoclast parameters. Unstained sections were used for fluorescent double label analysis and calculation of dynamic parameters of bone formation using a Nikon Eclipse E400 microscope equipped with fluorescent light, an Olympus DP73 camera and OsteoMeasure7 software (OsteoMetrics, Inc.). The abbreviations recommended by the American Society for Bone and Mineral Research Histomorphometric Nomenclature Committee (Dempster et al., 2013).

### Statistical analysis

Among the genotype groups, statistical comparisons for DEXA, micro-computed tomography and biomechanics were performed with one-way ANOVA when normality (Shapiro’s test) and homogeneity of variance (Levene’s test) were below 0.05 level of statistical significance. Otherwise, non-parametric test (Krustal-Wallis) were used. Post-hoc analysis was performed (Dunn’s test) for comparisons below 0.05 level of statistical significance, and *p*-values were adjusted for multiple comparisons.

## Results

### Generation of a mouse model to study SMC

To study the role of RAB33B in the skeleton we generated mice that carry a *Rab33b* missense pathogenic variant, c.136A>C (p.Lys46Gln), which was identified in members of a consanguineous family diagnosed with SMC (Alshammari et al., 2012). This variant replaces a highly conserved lysine within the GxxxxGK [S/T] guanine nucleotide-binding GTPase domain of RAB33B with a glutamine residue and in humans causes substantial protein loss in affected individuals (Alshammari et al., 2012). Using a CRISPR/Cas9 approach, we introduced the mutation into the mouse genome together with a few silent nucleotide changes just upstream of the Lysine 46 coding triplet, which were introduced to facilitate mouse genotyping (Figure 1). Four founder mice were generated and each of them was bred with a C57B6 mate. Offspring from each mating pair was genotyped by PCR and then the region of interest was sequenced (Sanger). Two males and two females (F1) were confirmed to be heterozygous for the desired mutation and were crossed to generate homozygous mice (Figure 1B). Homozygous *Rab33b* ^*A136C/A136C*^ mice were born at the expected Mendelian ratio and did not show macroscopic differences compared to their WT or heterozygous littermates.

### Effects of the *Rab33b* p.Lys46Gln disease variant at the cellular level

**FIGURE 2:**
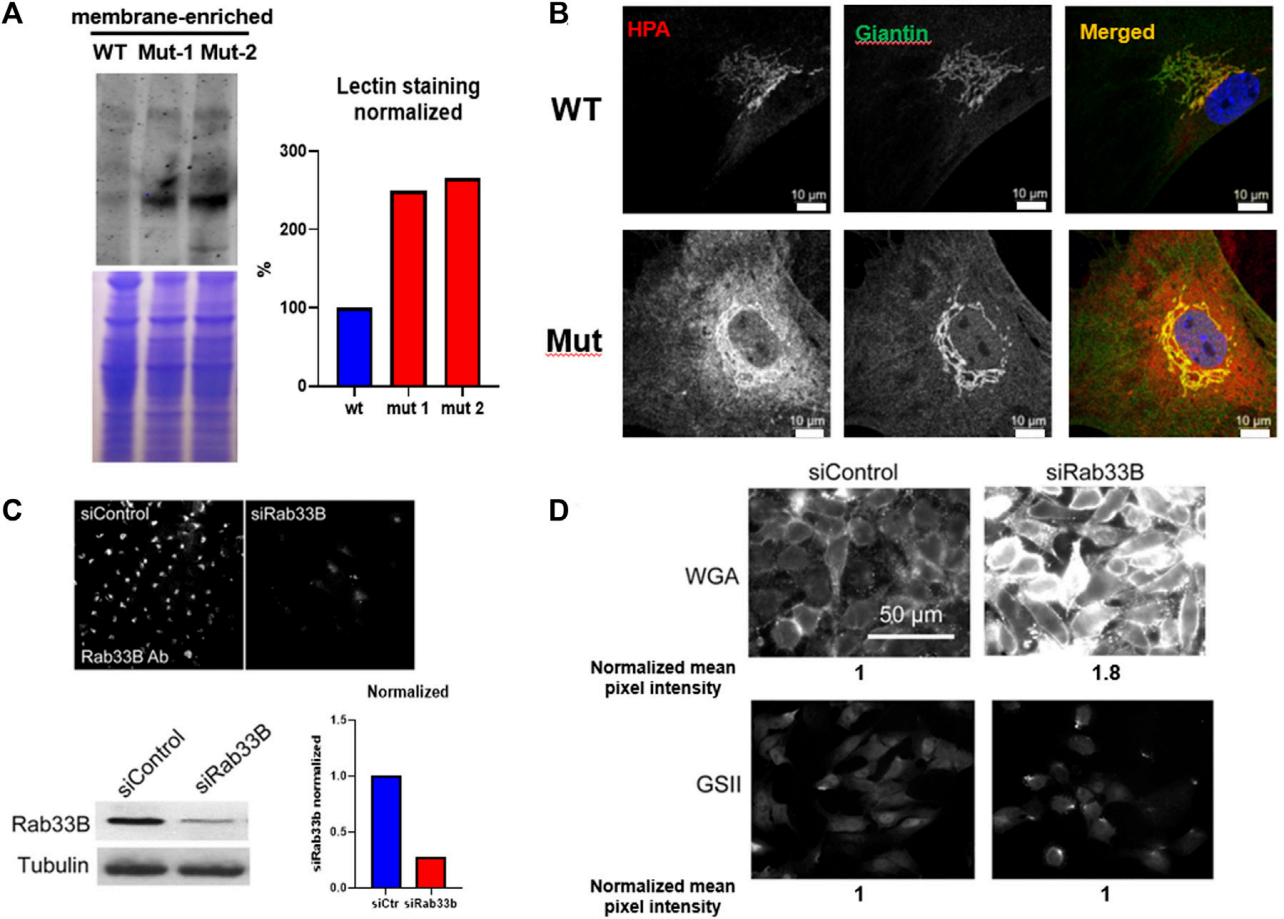
The *Rab33b* c.136A>C variant causes altered protein glycosylation. **(A)**. Western Blot using the same membrane-enriched lysates of freshly dissected adult liver shown in Figure 1, separated by SDS-PAGE and stained with the *Helix pomatia* agglutinin (HPA) lectin which selectively binds to α-N-acetylgalactosamine residues. The normalized lectin staining quantification is also shown. **(B)**. Immunofluorescence staining using HPA and the Golgi marker Giantin on primary calvarial osteoblasts and showing increased HPA staining inside the mutant osteoblasts. Scale bars = 10 μm **(C)**. Immunofluorescence and western blot on HeLa cells transfected with either control or siRNA against Rab33b (siRab33b), showing significantly reduced RAB33B protein levels as quantified in the graph. **(D)**. The same HeLa cells stained with WGA and GSII lectins. WGA staining was increased in cells transfected with siRab33b as quantified by normalized mean pixel intensity (average of 300 cells).

To study the effects of the p.Lys46Gln pathogenic variant on the level of expression of RAB33B protein, we collected livers from homozygous mutant and WT adult mice and performed Western blot analysis. The liver provides a ready source for a membrane-enriched preparation that makes it easier to detect membrane-associated proteins that are not expressed at high levels such as RAB33B. Western blot showed 40%–60% reduction of the RAB33B protein in homozygous mutant livers compared to a WT control, suggesting that the p.Lys46Gln variant has a hypomorphic effect and does not result in complete loss of protein expression in the mouse model (Figure 1C). See also Supplementary Figure S1 for a total liver lysate. This observation was confirmed using immunofluorescence on primary calvarial osteoblasts. In these cells we observed co-localization of the RAB33B protein with the Golgi marker Giantin in WT control cells but levels of RAB33B protein were significantly reduced in cells from homozygous mutant mice (Figure 1D). We then separated on a polyacrylamide gel the same membrane-enriched liver fractions used for the detection of the RAB33B protein, transferred proteins to the membrane, and stained with the fluorescently labelled *Helix pomatia* agglutinin (HPA), a lectin that selectively binds to α-N-acetylgalactosamine residues (Tn antigen), an intermediate glycan transiently accumulated during O-glycosylation of proteins in the Golgi. With some degree of variability, a few distinct bands showed significantly increased HPA staining in the homozygous mutant compared to the WT control lysates, indicating altered proteins glycosylation in liver tissue (Figure 2A). To confirm this finding in bone cells, we performed immunofluorescence on primary calvarial osteoblasts and, in some cells more than others, observed a modest increase in HPA staining in the homozygous mutant compared to WT osteoblasts (Figure 2B). To evaluate potential glycosylation defects in human cells, we used a short interference RNA approach to downregulate the expression of RAB33B in HeLa cells (Figure 2C). We then used these HeLa cells and stained them with multiple fluorescently labelled lectins, including *Wheat Germ Agglutinin* (WGA) and *Griffonia simplicifolia* (GSII). These lectins recognize terminal α-N-acetylglucosamine and sialic acid (WGA) or α-N-acetylglucosamine residues (GSII), common for protein N-glycosylation in the Golgi. While the GSII staining was not significantly different from control cells, HeLa cells that expressed less RAB33B showed a prominent increase in WGA staining compared to controls and quantified by normalized mean pixel intensity (Figure 2D), supporting the notion that RAB33B-deficient cells display altered protein glycosylation.

### Effects of the *Rab33b* p.Lys46Gln variant on the skeleton

**FIGURE 3:**
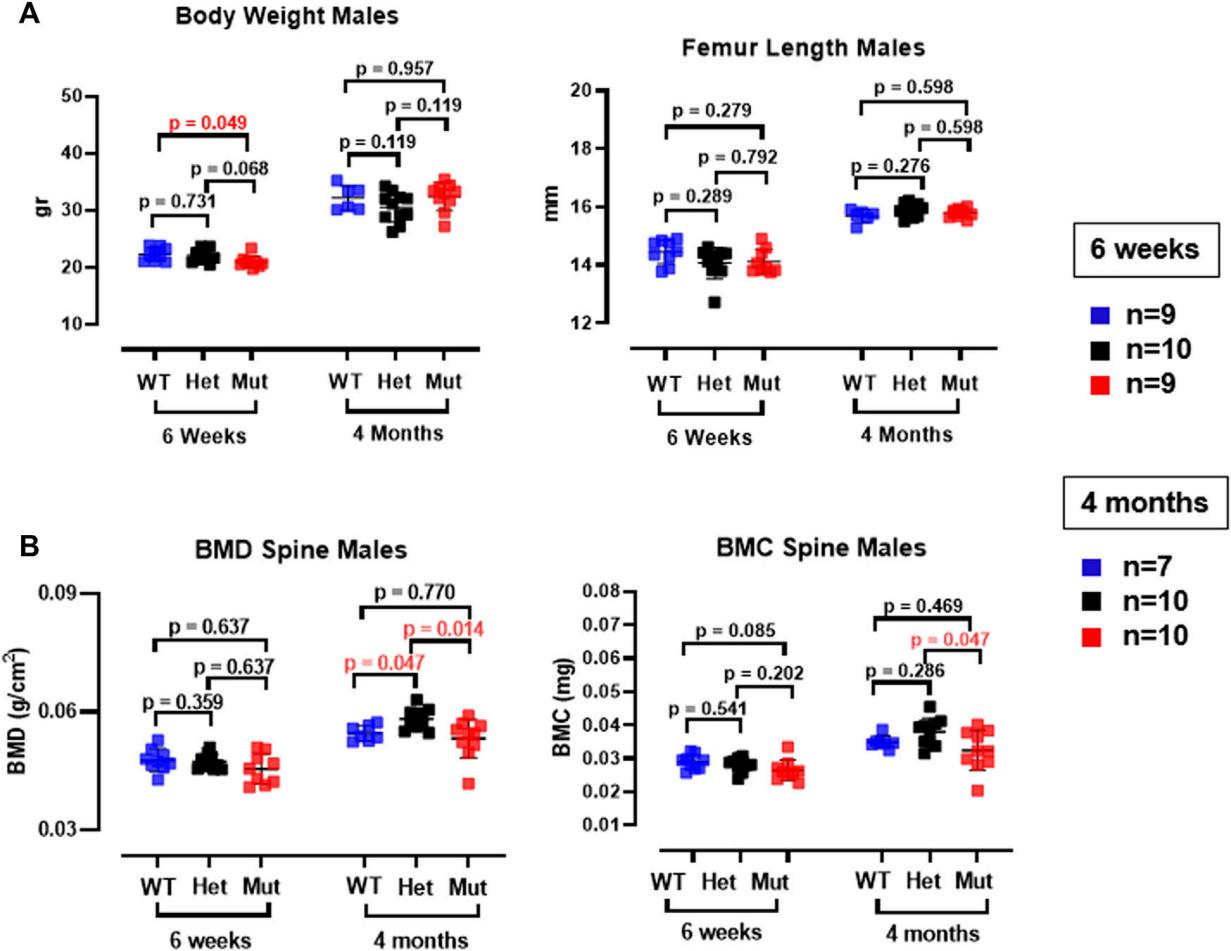
Body weight, femur length and DEXA measurements in *Rab33b* mutant male mice. **(A)**. Body weight and femur length were not different between heterozygous or homozygous and WT male mice at 6 weeks or 4 months of age. **(B)**. Lumbar spine bone mineral density (BMD) and bone mineral content (BMC) in male mice at 6 weeks and 4 months of age. Note the small but significant increase in the BMD in the spine in het mice.

**FIGURE 4:**
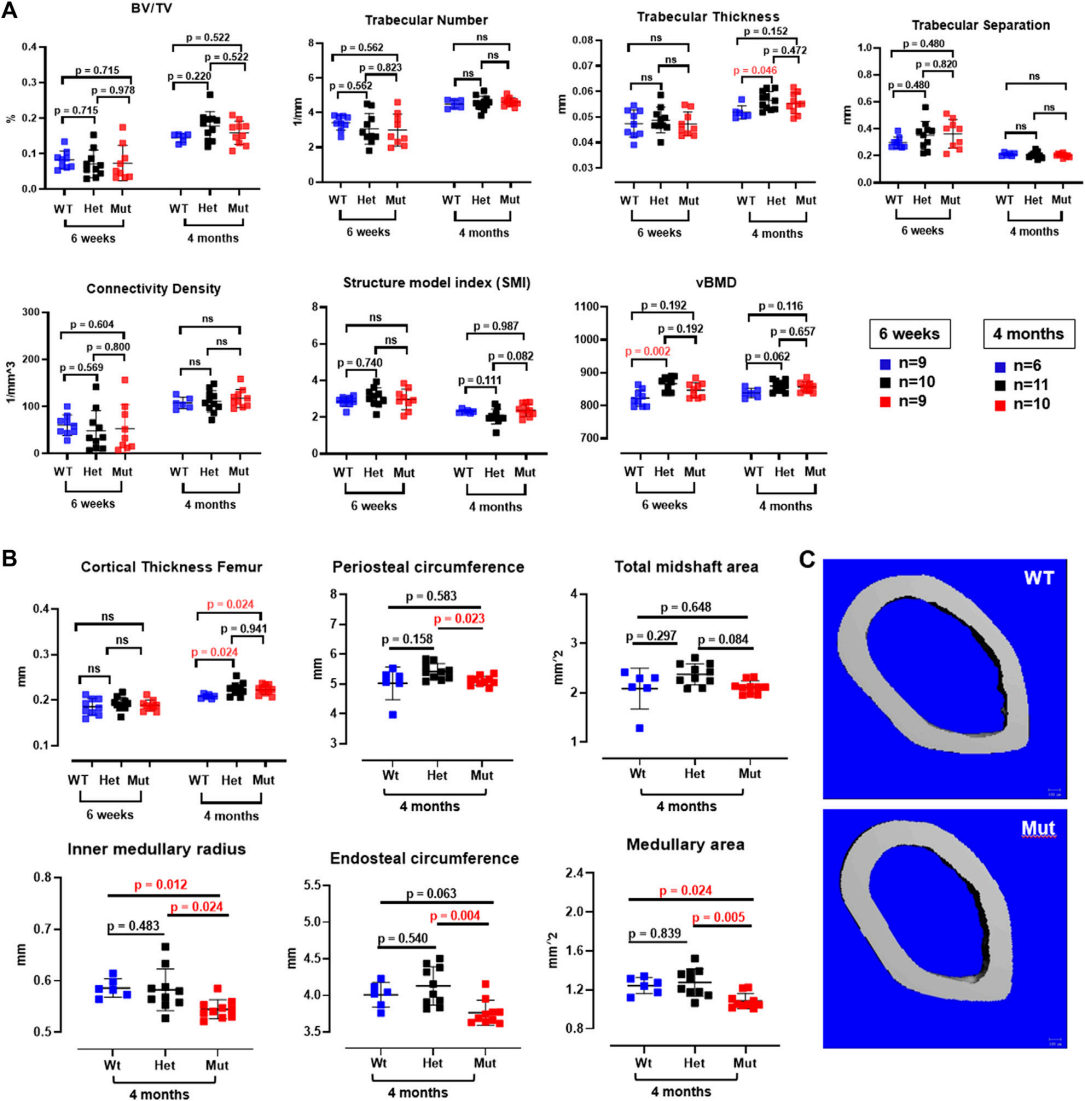
MicroCT analysis of male femurs. **(A)**. Trabecular bone parameters in the femur were measured at both 6 weeks and 4 months of age in all mouse genotypes. Trabecular thickness was elevated at 4 months of age in heterozygous *Rab33b* mice. vBMD was also higher in het mice at 6 weeks of age. **(B)**. Cortical bone parameters at the femur midshaft showed increased cortical thickness at 4 months in both heterozygous and homozygous *Rab33b* mutant mice. While total midshaft area and periosteal circumference were not different from control mice, homozygous Rab33b mutant mice had decreased medullary area, endosteal circumference and inner medullary radius compared to heterozygous and WT mice. **(C)**. Representative microCT 3D-rendering of the femur midshaft from a control and homozygous mutant mouse.

**FIGURE 5:**
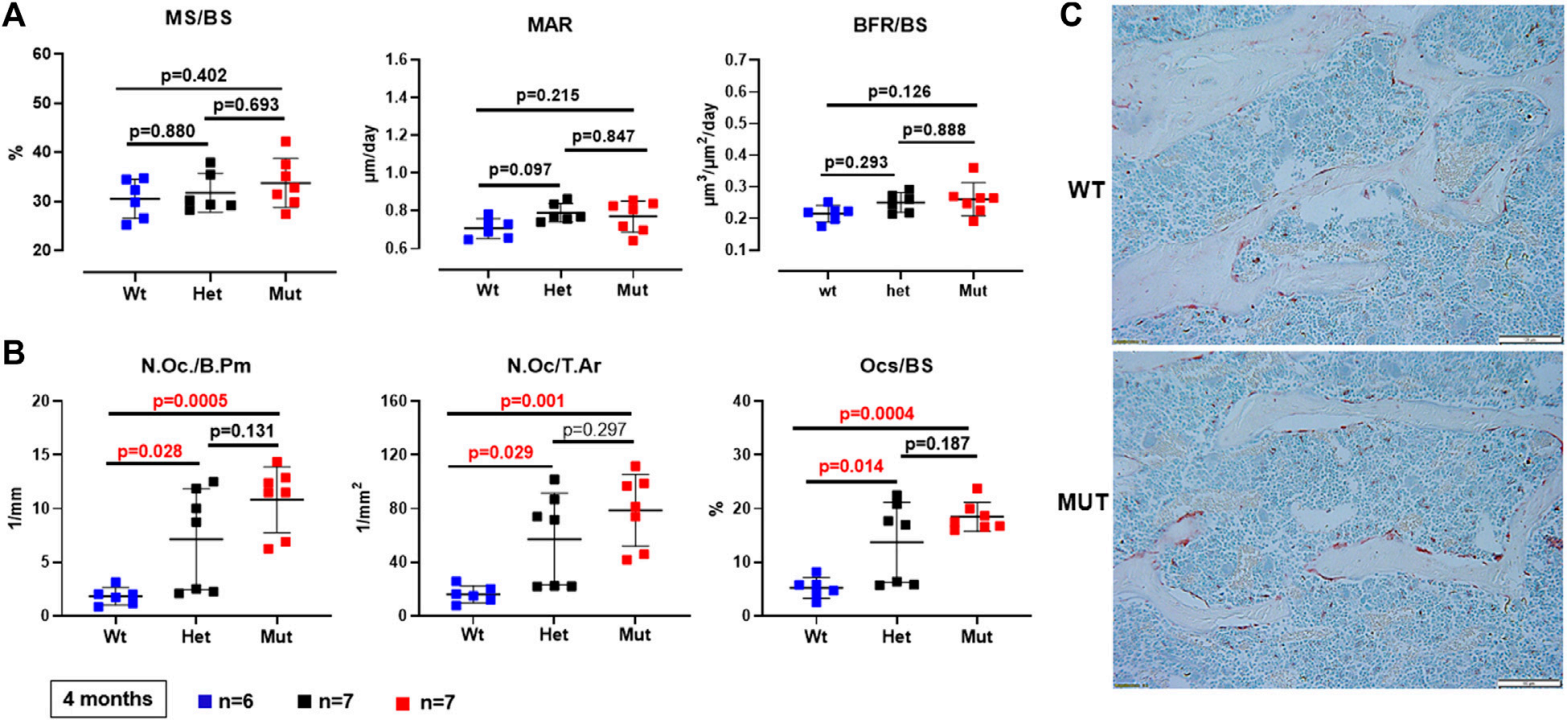
Bone histomorphometry of the lumbar spine (L4). **(A)**. Dynamic parameters of bone formation (MS/BS = mineralizing surfaces/bone surfaces; MAR = mineral apposition rate; BFR/BS = bone formation rate/bone surfaces) were not significantly different among genotypes. **(B)**. Osteoclast parameters (N.Oc/B.Pm = number of osteoclasts/bone perimeter; N.Oc/T.Ar = number of osteoclasts/tissue area; OcS/BS = osteoclast surface/bone surface) were increasingly elevated in heterozygous and homozygous compared to WT mice. **(C)**. Representative images showing trabecular bone within a vertebral section and TRAP-positive (Tartrate-resistant acid phosphatase) osteoclasts from a WT and a homozygous mutant *Rab33b* mouse.

Because the effects of the p.Lys46Gln variant in the mouse were unknown and difficult to predict across species, we decided to study the skeleton of both heterozygous and homozygous mutant mice. We did this in actively growing 6 weeks old mice and in fully grown, young adult mice at 4 months (16 weeks) of age, which have a fully mature skeleton. Other than a small (decreased) but significant difference in body weight in homozygous mutant compared to WT males at 6 weeks of age, body weight and femur lengths were not different among male or female mice at either age (Figure 3A and data not shown). Dual energy absorptiometry (DEXA) to determine their bone mineral density (BMD) and bone mineral content (BMC) showed a mild increase in BMD in the lumbar spine of heterozygous male mice at 4 months (*p* = 0.047 vs. WT) (Figure 3B). No differences were noted in young males or in female mice of either age (Figure 3B and Supplementary Figure S2). Micro-computed tomography (microCT) showed no differences in bone volume/tissue volume (BV/TV) or trabecular bone number (Tb.N) in the lumbar spine, but the bone trabecular thickness (Tb.Th) was significantly higher in heterozygous 4 month-old males compared to WT (*p* = 0.024 vs. WT) (Supplementary Figure S3). Other parameters measured in the spine were not different. No differences were noted in the spine of 6-week-old females (Supplementary Figure S4) and no additional microCT analyses were performed in females. The analysis of distal femurs in male mice confirmed the increased trabecular thickness observed in the spine, but again only in 4 months old heterozygous mice (*p* = 0.046 vs. WT) (Figure 4). Femoral volumetric bone mineral density (vBMD) was elevated at both 6 weeks and 4 months of age in heterozygous males vs. WT (*p* = 0.002 and *p* = 0.062, respectively) (Figure 4A). Additional mid-shaft femoral cortical parameters showed a significant increase in cortical thickness in both heterozygous and homozygous male mice at 4 months (*p* = 0.024 each vs. WT) (Figure 4B). While the periosteal circumference and total mid-shaft area (cross sectional area) were not different compared to control mice, indicating bones of similar size and diameter, both the inner medullary radius and endosteal circumference were significantly smaller in homozygous male mice at 4 months of age which resulted in a decreased medullary area (Figures 4B,C). Overall, the increased trabecular and cortical thickness and reduced medullary area at 4 months in Rab33b mutant mice suggested a potential reduction in bone resorption. To evaluate for changes at the bone cellular level that could explain these observations, we performed bone histomorphometry on L4 vertebral bodies (lumbar spine) in 4-month-old male mice. Dynamic parameters of bone formation, including mineralizing surfaces over bone surfaces (MS/BS), mineral apposition rate (MAR) and bone formation rate (BFR/BS) trended, on average, to be mildly elevated but without reaching the significance level (Figure 5A). Conversely and surprisingly, osteoclast numbers (N.Oc./B.Pm and N.Oc./T.Ar) and surfaces (Ocs/BS) were about 4 folds higher in the homozygous mutant compared to WT spines, with heterozygous mice showing a similar, though less pronounced, elevation of the osteoclast parameters compared to WT mice (Figures 5B,C). An *in vitro* osteoclast differentiation assay using bone marrow macrophages from WT and *Rab33b* mutant mice showed increased osteoclastogenesis, consistent with what we observed *in vivo* (Supplementary Figure S5). The finding of elevated osteoclasts parameters in spite of increased trabecular and cortical thickness suggested that these cells are not able to properly resorb bone and pointed to a putative functional important role for RAB33B in this cell lineage.

### Biomechanical assessment of Rab33b mutant femurs

**FIGURE 6:**
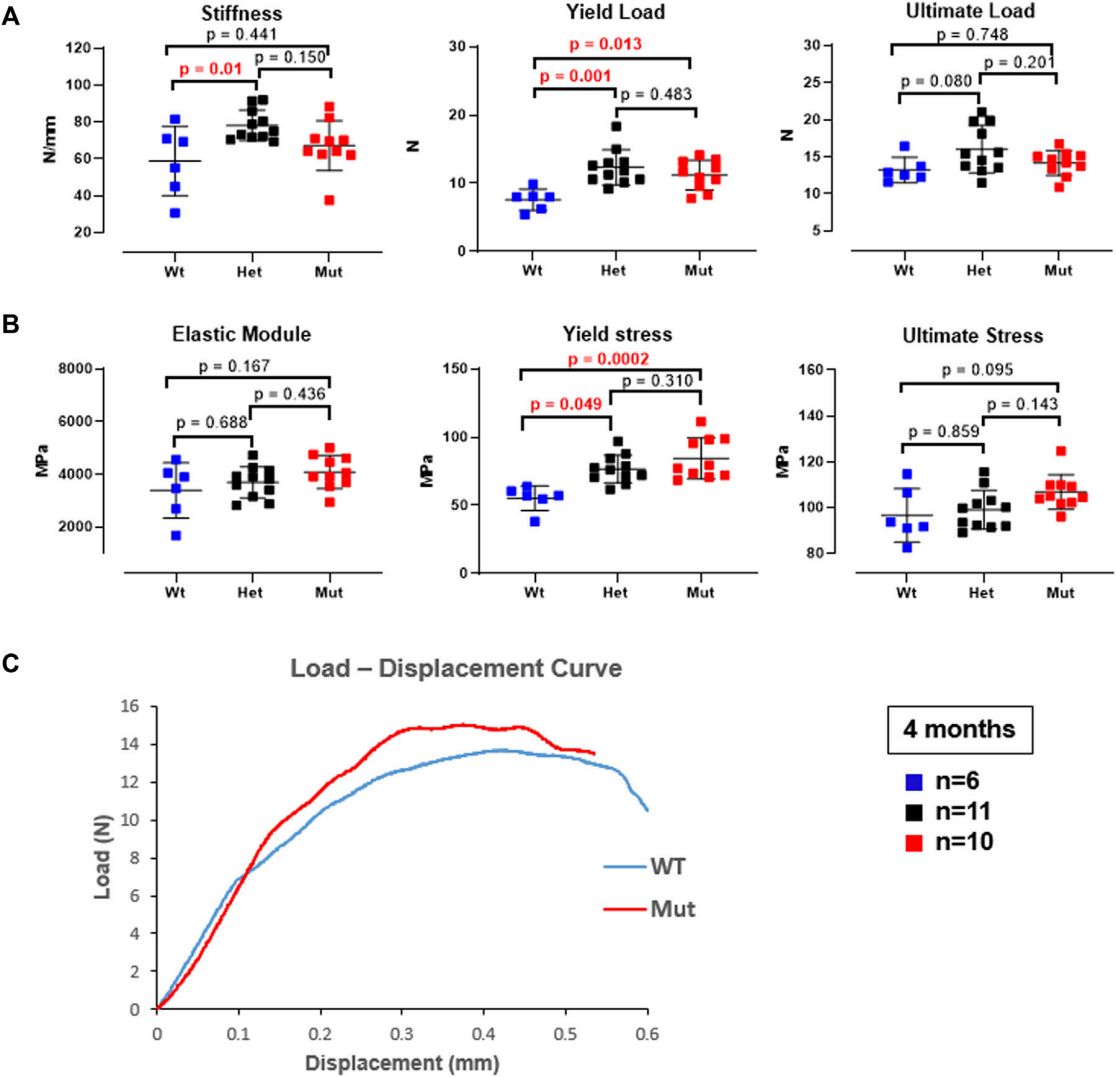
Three-point bending biomechanical test on femurs from 4-month-old male mice. **(A)**. Bone mechanical properties, including stiffness and yield load were elevated in het mice and the yield load was also elevated in mut mice compared to controls. **(B)**. The corresponding estimated material properties, including Elastic modulus, yield stress, and ultimate stress trended progressively higher and reached significance for the yield stress in both het and mutant compared to WT control mice. **(C)**. Representative load-displacement curve from a homozygous mutant and a WT control mouse.

The higher femoral volumetric bone mineral density and cortical thickness suggested that *Rab33b* mutant mice may have altered biomechanical bone properties compared to controls. To assess this, we next performed a femoral three-point bending structural mechanical test in 4 month-old male mice. Standard mechanical properties derived from this test identified a significantly elevated yield load (yield force) in both heterozygous and homozygous mice compared to controls (*p* = 0.001 and *p* = 0.013, respectively). Stiffness was also significantly elevated but only in heterozygous males (*p* = 0.01 vs. WT) (Figure 6A). Interestingly, the calculation of the corresponding estimated material properties such as Young’s modulus (Elastic Modulus), yield stress and ultimate stress showed a progressive elevation of these parameters from WT to heterozygous to homozygous mutant mice, although only the yield stress was significantly different in homozygous and heterozygous compared to WT mice (*p* = 0.0002 and *p* = 0.049, respectively) (Figure 6B). A representative load-displacement curve comparing WT vs. homozygous male mice is shown in Figure 6C.

### Histology and lectin staining on bone tissue sections

**FIGURE 7:**
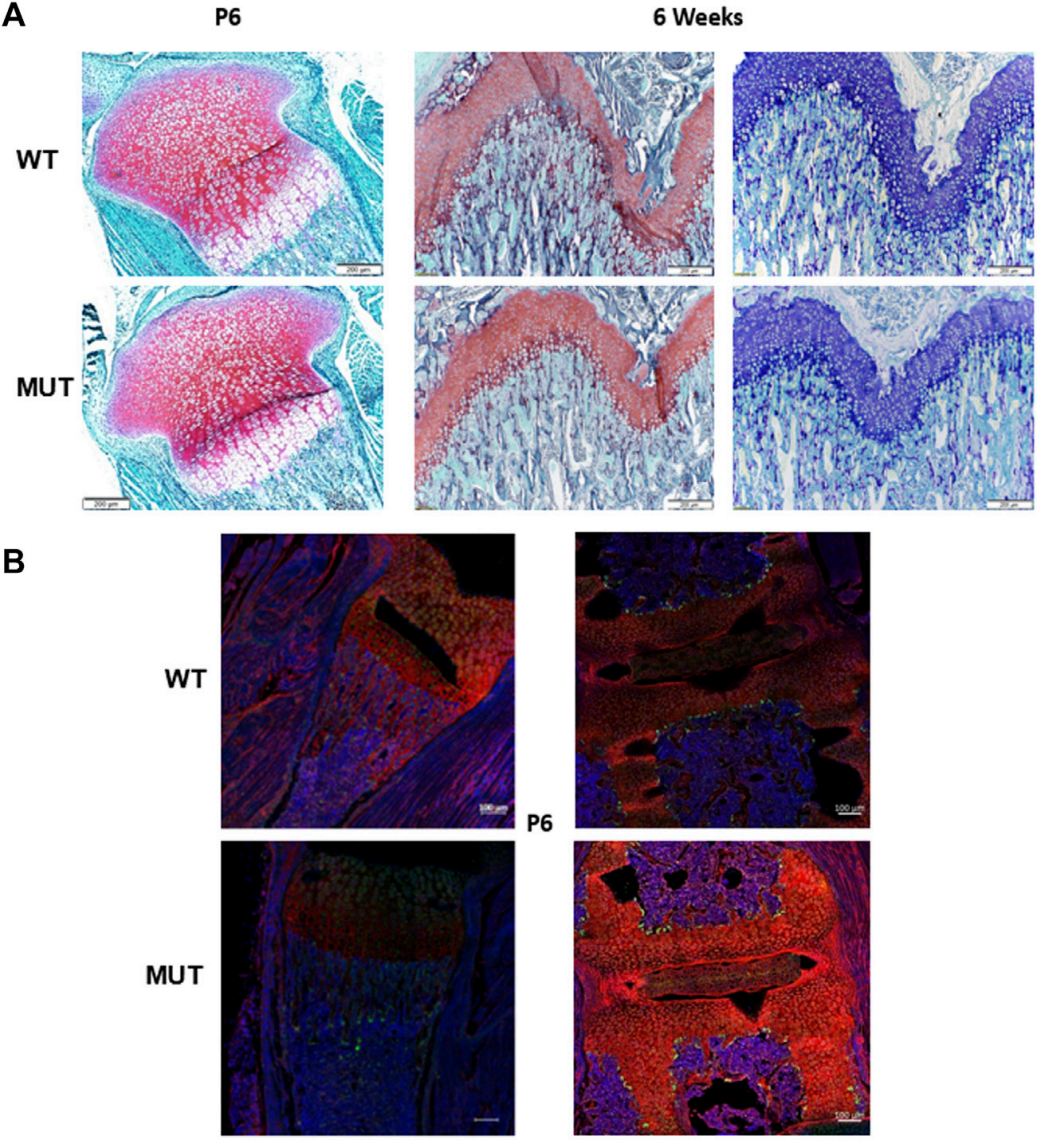
Histology and immunofluorescence staining. **(A)**. Safranin-O and Alcian blue staining of tibia growth plates from 6 days old and 6 weeks old showed no macroscopic differences between homozygous mutant and WT control mice. **(B)**. Qualitative WGA (red) and GNL (green) lectin immunofluorescence staining in proximal tibia (left) and spine (right) at P6 showed variable and often inconsistent results between sections from homozygous mutant and WT control mice.

Mild alterations in growth plate and cartilage histology were reported earlier due to mutations in Dymeclin (Osipovich et al., 2008). To determine if the growth plate morphology was affected in *Rab33b* mutant mice, we performed histology of distal femurs and proximal tibias in 6-day-old and 6-week-old mice and stained sections with Safranin-o and Alcian Blue for a qualitative assessment of proteoglycan expression. Neither the morphology of the growth plate nor the staining with Safranin-o and/or Alcian Blue revealed any striking differences between homozygous and WT mutant mice (Figure 7A). Next, to assess the potential difference in the expression of specific cellular sugar residues on these tissue sections, we proceeded to stain 6-day-old sections (derived from n = 2 WT and n = 3 homozygous mutants) with 2 fluorescently labeled lectins, Wheat Germ Agglutinin (WGA) and *Galanthus Nivalis Lectin* (GNL) and acquired immunofluorescence light images at the confocal microscope. GNL recognizes terminal mannose residues in immature N-glycosylated proteins. We observed differences between WT and Rab33b homozygous mutant samples but with significant variability among samples of different genotypes and also within the same genotype using identical acquisition parameters (Figure 7B).

### X-ray images of the skeleton

**FIGURE 8:**
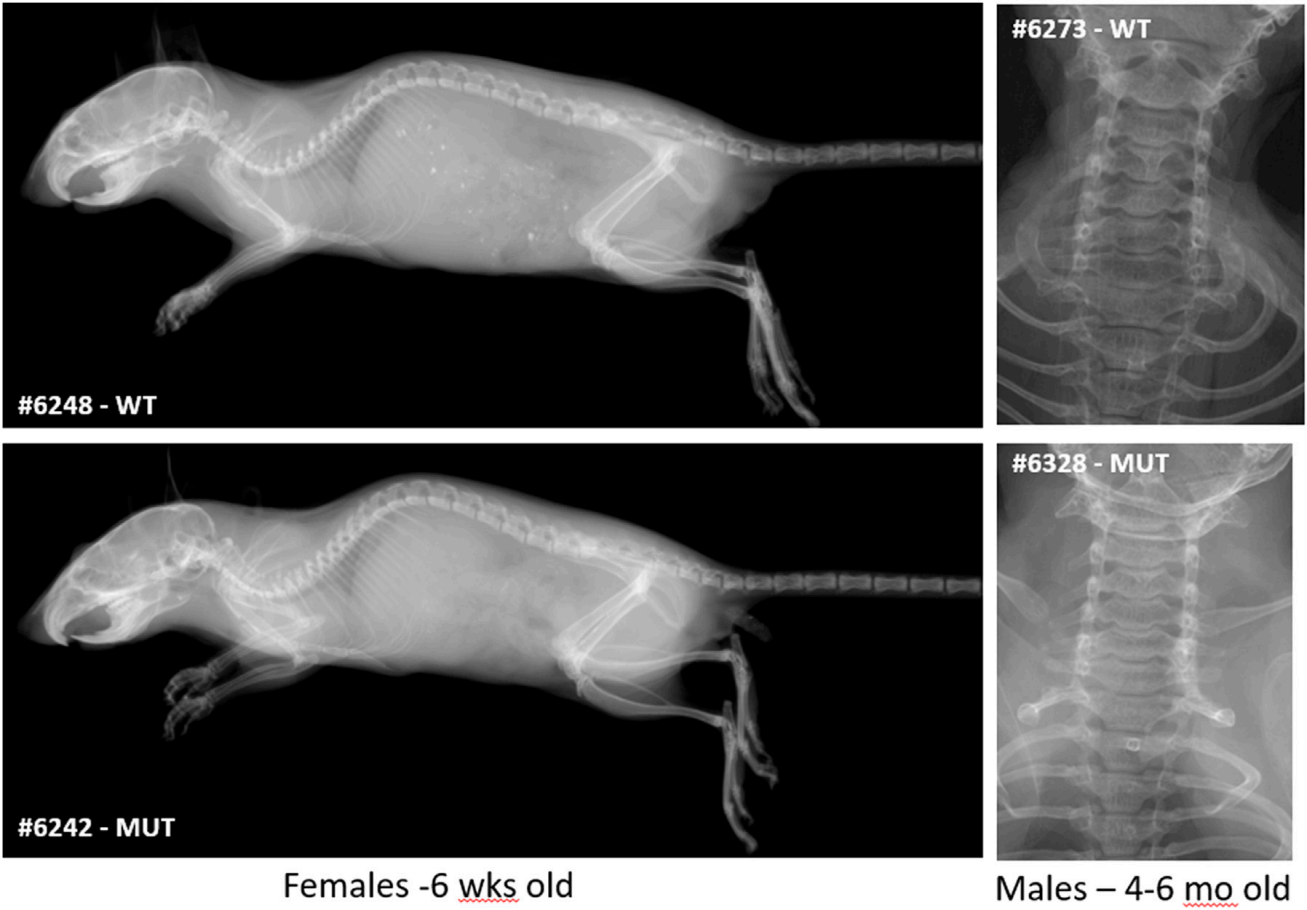
Skeletal survey. X-ray images at either 6 weeks (full skeleton) or 4–6 months of age (upper thoracic and neck area) did not show significant macroscopic differences or cervical vertebral malformations typical of SMC2.

Finally, because SMC patients show a pathognomonic malformation of vertebrae, characterized by double-humped vertebral bodies and platyspondyly, causing short neck and trunk with barrel-shaped chest, we carefully analyzed the skeleton of *Rab33b* mutant mice to determine if this mouse model reproduced similar vertebral morphological defects. Digital X-ray images acquired either at 6 weeks of age or between 4 and 6 months of age did not show any atypical skeletal malformation in either heterozygous or homozygous mice compared to WT controls (Figure 8).

## Discussion

The disease mechanisms causing Smith-McCort skeletal dysplasia, due to *RAB33B* pathogenic variants, are not well understood. In this study we generated mice that carry a *Rab33b* disease-causing variant, c.136A>C (p.Lys46Gln), which is identical to that of members from a consanguineous family diagnosed with SMC2, and studied their skeleton. The *Rab33b* variant caused a mild increase in trabecular bone thickness in the spine and femur and in femoral mid-shaft cortical thickness with a concomitant reduction of the femoral medullary area at 4 months of age. Since we found no differences in dynamic parameters of bone formation in mutant *versus* control mice, we sought to investigate a potential reduction in trabecular and endosteal bone resorption activity. However, bone histomorphometry unexpectedly showed a 4-fold increase in osteoclast parameters in homozygous *Rab33b* mice. Because the increased number of osteoclast cells did not translate in reduced bone mass, it suggested a putative impairment of bone resorption and a potential novel role of RAB33B in osteoclast function which needs to be further explored. Femur three-point bending biomechanical tests showed an increased in yield load and a progressive elevation, from WT to heterozygote to homozygous mutants, of all bone intrinsic properties. These findings indicate an overall impact on bone material properties which may be caused by disturbed protein glycosylation in cells contributing to skeletal formation, as suggested by the altered and variable pattern of lectin staining. Overall, the phenotype of either heterozygous or homozygous mice was subtle, did not reproduce some of the typical features of the human disease and was also sex-specific, manifesting in males but not females.

From our work, two aspects are emerging on the role of RAB33B in the skeleton. The first is the novel potential role of RAB33B in osteoclast function; the second is its role at the Golgi apparatus to allow correct protein processing and glycosylation. Our findings add more evidence to the important role that several RAB proteins play in osteoclast biology and bone resorption (for a review, see (Roy and Roux, 2018; Roy and Roux, 2020)). For instance, the downregulation of RAB7 was shown to impair the formation of the osteoclast F-actin ring and the process of osteoclast polarization, causing a severe reduction in bone resorption (Zhao et al., 2001). Importantly, mutations in an effector of RAB7, PLEKHM1, cause a form of osteopetrosis in humans (Van Wesenbeeck et al., 2007; Bo et al., 2016). Several other Rab-GTPases, their regulators, GEFs and GAPs, and effectors are expressed in osteoclasts and although their role is not fully understood, they are believed to take part in endosomal trafficking processes that are important for the formation of the osteoclast ruffle border, a key structure for bone resorption, but are also involved in the process of autophagy (Zhao et al., 2002; Roy and Roux, 2018; Roy and Roux, 2020). Autophagy in osteoclasts can promote podosome disassembly and thus facilitate cell motility (Zhang et al., 2020). *RAB33B* is expressed in human osteoclasts (Roy and Roux, 2020) and in non-osteoclastic cells, it is also involved in autophagosome formation and maturation (Itoh et al., 2008; Itoh et al., 2011; Morgan et al., 2019). In addition, a recent siRNA screen to identify RAB proteins involved in cell migration, identified RAB33B as a strong candidate in the regulation of focal adhesion dynamics by modulating the delivery of integrins to focal adhesions (Bjornestad et al., 2022). Therefore, RAB33B could be important in numerous aspects of osteoclast biology. These range from the establishment of the actin ring, a specialized adhesion structure that osteoclasts coordinate to seal onto the bone surface, to the genesis of the ruffled border, to the autophagosome formation and potentially, osteoclast motility. These processes warrant future investigations, preferably in the context of a complete loss of function of RAB33B in primary osteoclast cells.

Our findings in the Golgi apparatus suggested that disruption of RAB33B function results in altered protein glycosylation, likely due to its role in normal vesicular trafficking at this organelle. Although we encountered significant variability in the lectin staining assays that we performed on murine primary cells and tissues, primary RAB33B mutant osteoblasts as well as liver lysates appeared to have increased staining with Helix pomatia agglutinin (HPA) which binds to N-acetylgalactosamine residues. This indicates alterations in protein O-glycosylation compared to WT controls. Furthermore, data from HeLa cells in which RAB33B expression was significantly reduced by siRNA, showed a clear increase in WGA staining compared to control cells, supporting the link between RAB33B function and Golgi glycosylation. A disturbed protein glycosylation process was also indicated by the altered and contrasting staining with WGA in RAB33B mutant 6-day old embryo sections of long bones and spines compared to WT. WGA binds N-acetylglucosamine and sialic acid residues which are sugar structures common to many membrane proteins. Conversely, staining with GNL which binds to mannose residues was mostly unchanged in mutant compared to WT mice. Changes in protein glycosylation can affect many proteins, both intracellular as well as those that are membrane-bound or secreted. Osteoblasts, in addition to type I collagen, secrete several other proteins that are either components of the extracellular matrix (ECM) or bind to the ECM, e.g., osteocalcin, and some of them are known to regulate the matrix mineralization process. The changes that we observed in 3-pt bending tests of the femurs showed altered bone material properties which are indeed consistent with a likely effect of altered protein glycosylation onto bone accrual and mineralization. Such changes resulted in an increased yield load and yield stress and indicate that the mutant bones required more force before beginning to irreversibly bend. These data, together with the slight increase in ultimate load and ultimate stress, suggest that intrinsically, *Rab33b* mutant bones may be stronger compared to WT bones. Interestingly, among all the typical features that SMC2 patients may present (Alshammari et al., 2012; Dupuis et al., 2013; Tuysuz et al., 2021), skeletal fractures are not one of them which supports our finding. Finally, changes in protein glycosylation have also been showed to impact mesenchymal stem cell differentiation and ultimately, the function of osteoblasts (Wilson et al., 2018). Therefore, future studies will also need to assess such potential outcomes in a *Rab33b* loss of function model.

Our work has some limitations. A study in primary cells derived from SMC2 patients indicated that, while the p.Lys46Gln variant is expected to impair the GTPase activity, it also severely reduced RAB33B protein levels (Alshammari et al., 2012), suggesting that the disease phenotype may result from a combination of the two effects. Conversely, the same amino acid change in the murine model only caused about 40%–60% reduction of RAB33B protein levels in homozygous mice and yielded a mild phenotype with a poor representation of the clinical features of the human disease. This perhaps indicates that RAB33B protein levels are also important since a residual enzymatic activity may, in part, compensate for the effects of the mutation. The most common clinical findings in SMC patients, including short neck and trunk, platyspondyly, hyperlordosis, various joint abnormalities, and short bones allude to a potential defect in endochondral ossification during growth, perhaps also mediated by a chondrocyte defect, which is not reproduced by our mouse model. The indication that protein glycosylation is affected in the mouse is an important finding and justifies future follow-up studies using a comprehensive mass-spectrometry glycomic approach in a complete loss of function model of *Rab33b* to better comprehend the underlying disease mechanism. The hypomorphic nature of the p.Lys46Gln variant in the mouse could also have contributed to the phenotype variability that we observed both *in vitro* and *ex vivo* with the lectin staining. The murine model may also be less sensitive to the effects of the p.Lys46Gln variant compared to humans. This is not an uncommon observation in mouse models of human disease (e.g. (Chen et al., 1998)). The outcomes of a complete *Rab33b* knockout remain to be tested. Furthermore, potential compensatory effects of the closely related paralog gene, *Rab33a*, could not be excluded and may be more prominent in the mouse compared to humans (see (Homma et al., 2021) for a phylogenetic tree of RABS). It is also interesting to note that, while the effects of the *Rab33b* variant were subtle, they were only detected in male (both heterozygous and homozygous) and not female mice. While we don’t have a current explanation for this sex difference, a total of fifteen SMC2 patients (9 females) have currently been described in the literature and we found no reference that the disease presents earlier and/or with more severity in males *versus* females (Alshammari et al., 2012; Dupuis et al., 2013; Salian et al., 2017; Tuysuz et al., 2021). This aspect may require further attention by clinicians.

In conclusion, as the Golgi apparatus is central to both secretory cargo transport and protein and lipid glycosylation, future studies of the effects of *Rab33b* pathogenic variants on the skeleton will provide additional opportunities to determine whether these are caused by a failure in cargo transport, a failure in glycosylation or a combination of both. Distinct bands in gel suggest strong effects on a limited set of proteins. The identification of specific cargos and/or glycosylation protein targets in both osteoclasts and osteoblasts will be important to further improve our understanding of RAB33B and Smith-McCort dysplasia.

## Data Availability

The original contributions presented in the study are included in the article/Supplementary Material S1, further inquiries can be directed to the corresponding author.

## Supplementary material

SUPPLEMENTARY FIGURE S1 Western Blot using a RAB33B antibody on membrane-enriched and total lysates of freshly dissected adult liver from 1 wild-type (WT) and 2 *Rab33b* mutant mice, separated by SDS-PAGE. Relative quantification of the bands is shown below the stained membrane.

SUPPLEMENTARY FIGURE S2 DEXA measurements in Rab33b mutant female mice at 6 weeks and 4 months of age. No significant differences were detected other than a small increase in BMC in the spine of mutant compared to het but not to WT controls.

SUPPLEMENTARY FIGURE S3 MicroCT analysis of male lumbar spines. Trabecular bone parameters in the spine were measured at both 6 weeks and 4 months of age in all mouse genotypes. Trabecular thickness was elevated at 4 months of age in both heterozygous and homozygous *Rab33b* mutant mice. BV/TV = bone volume/tissue volume; Tb-N = trabecular number; Tb-Th = trabecular thickness; Tb-Sp = trabecular separation; Conn density = connectivity density; SMI = structure model index; vBMD = volumetric bone mineral density.

SUPPLEMENTARY FIGURE S4 MicroCT analysis of female lumbar spines at 6 weeks of age. No significant differences were detected.

SUPPLEMENTARY FIGURE S5 Osteoclastogenesis assay. Trap-positive osteoclasts derived from bone marrow macrophages extracted from long bones of WT and homozygous mutant mice (n=1 – in 5 technical replicates) and treated with M-CSF and RANKL. The lower panels show higher magnification of yellow boxed areas.
