# Supplementary figures and images for: A Rab33b missense mouse model for Smith-McCort dysplasia shows bone resorption defects and altered protein glycosylation
Source: Front Genet. 2023 Jun 8;14:1204296. doi: 10.3389/fgene.2023.1204296 (PMC10285484; doi:10.3389/fgene.2023.1204296)

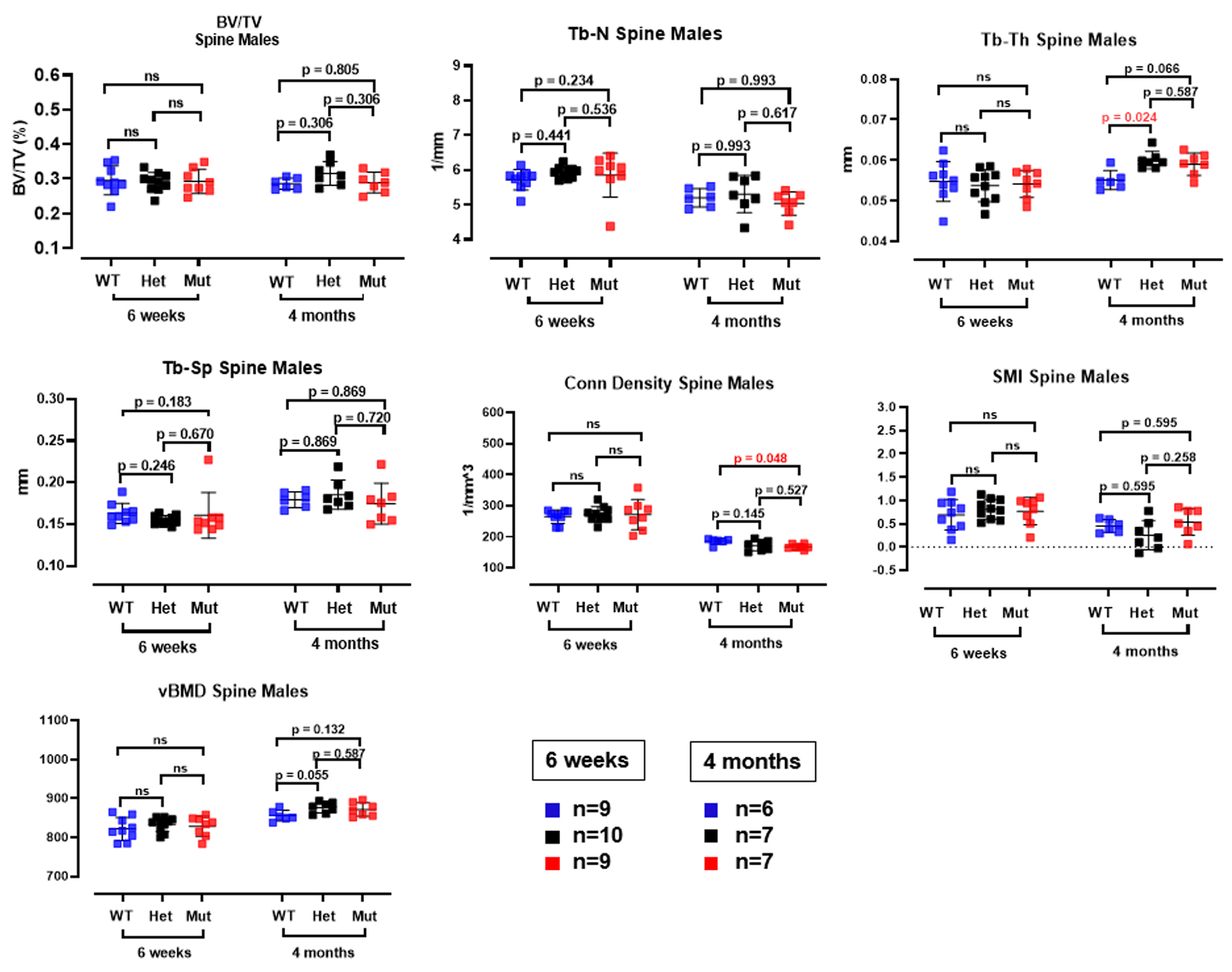

Supplement: Supplementary file 1 [file Image3.JPEG]

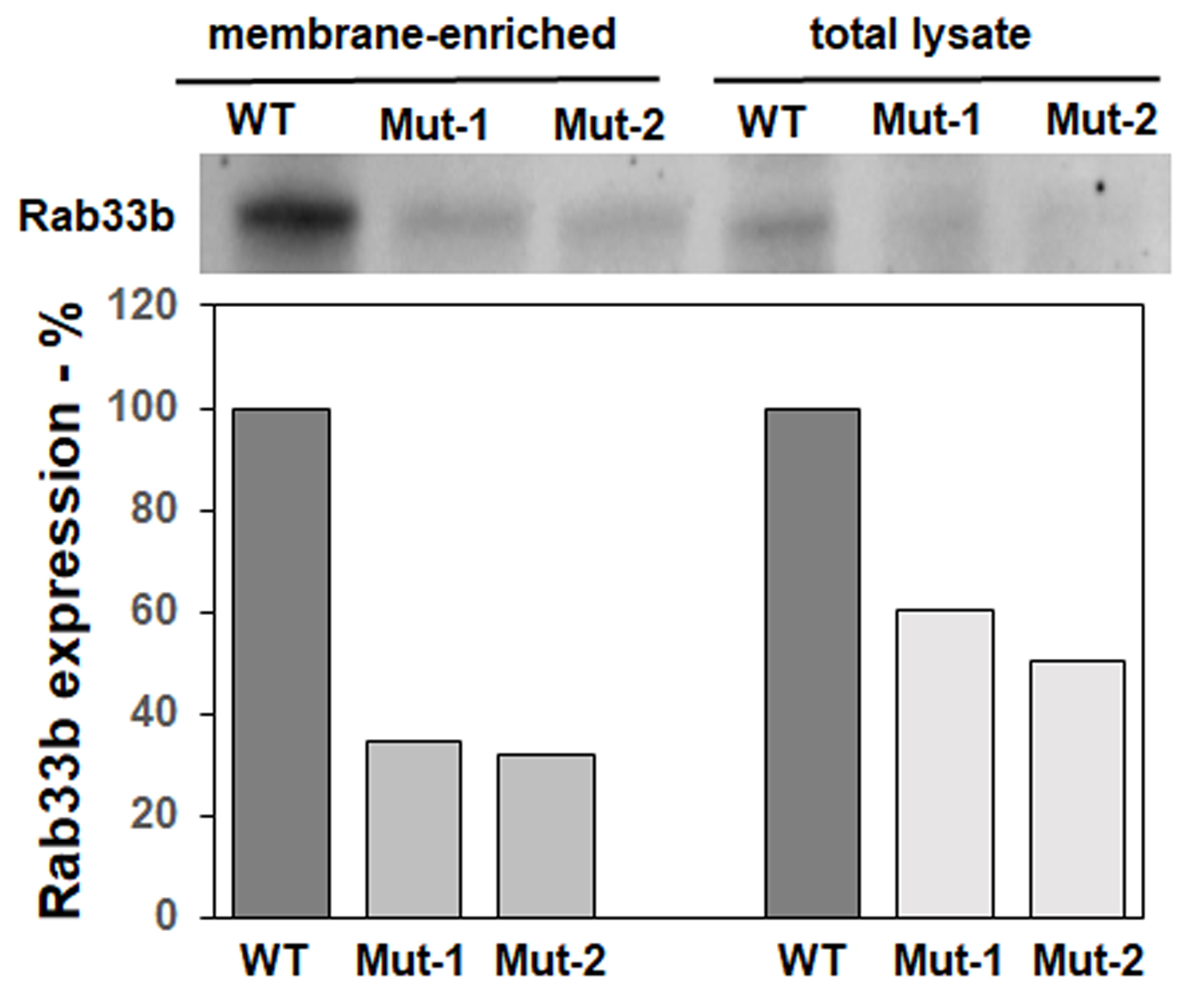

Supplement: Supplementary file 3 [file Image1.JPEG]

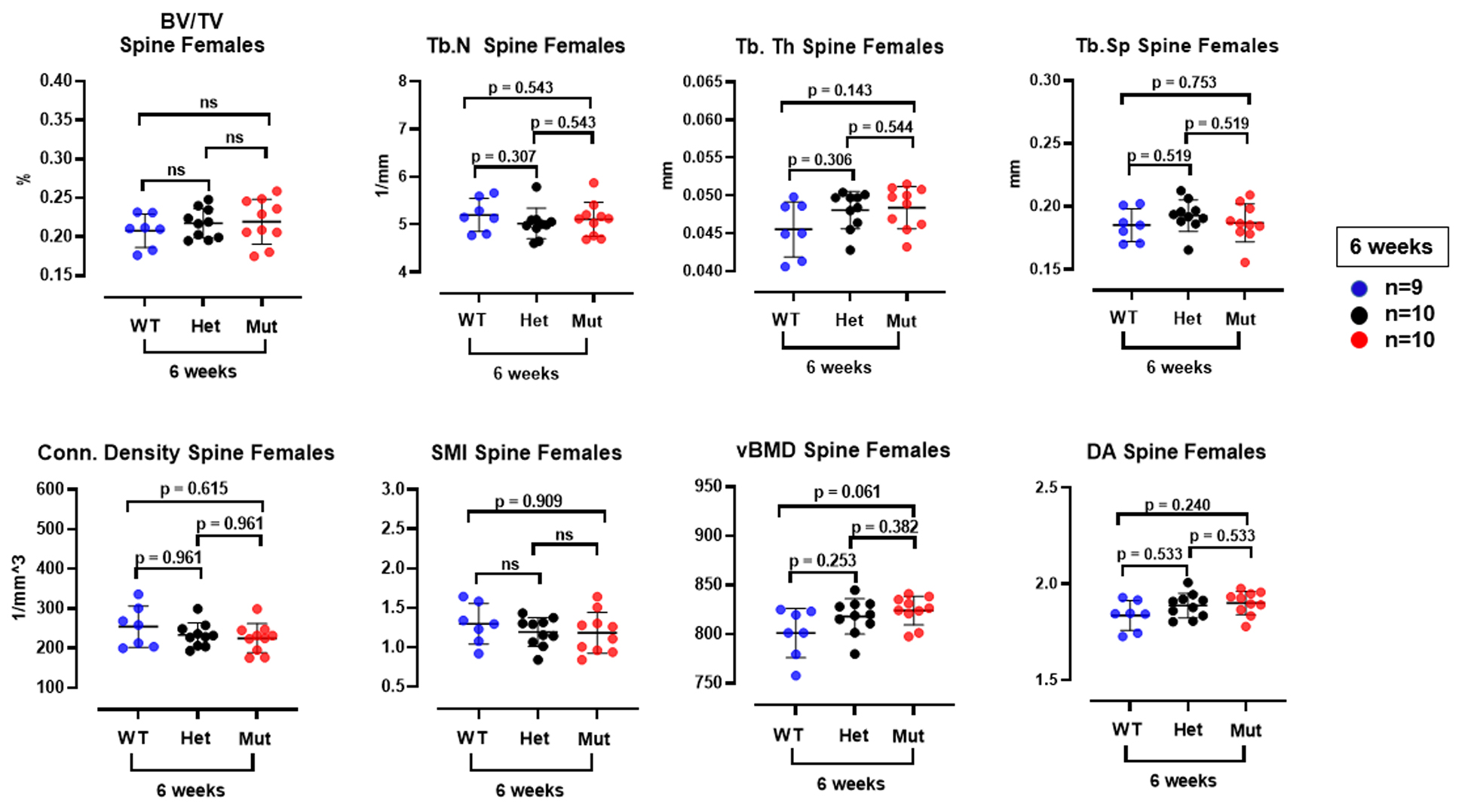

Supplement: Supplementary file 4 [file Image4.JPEG]

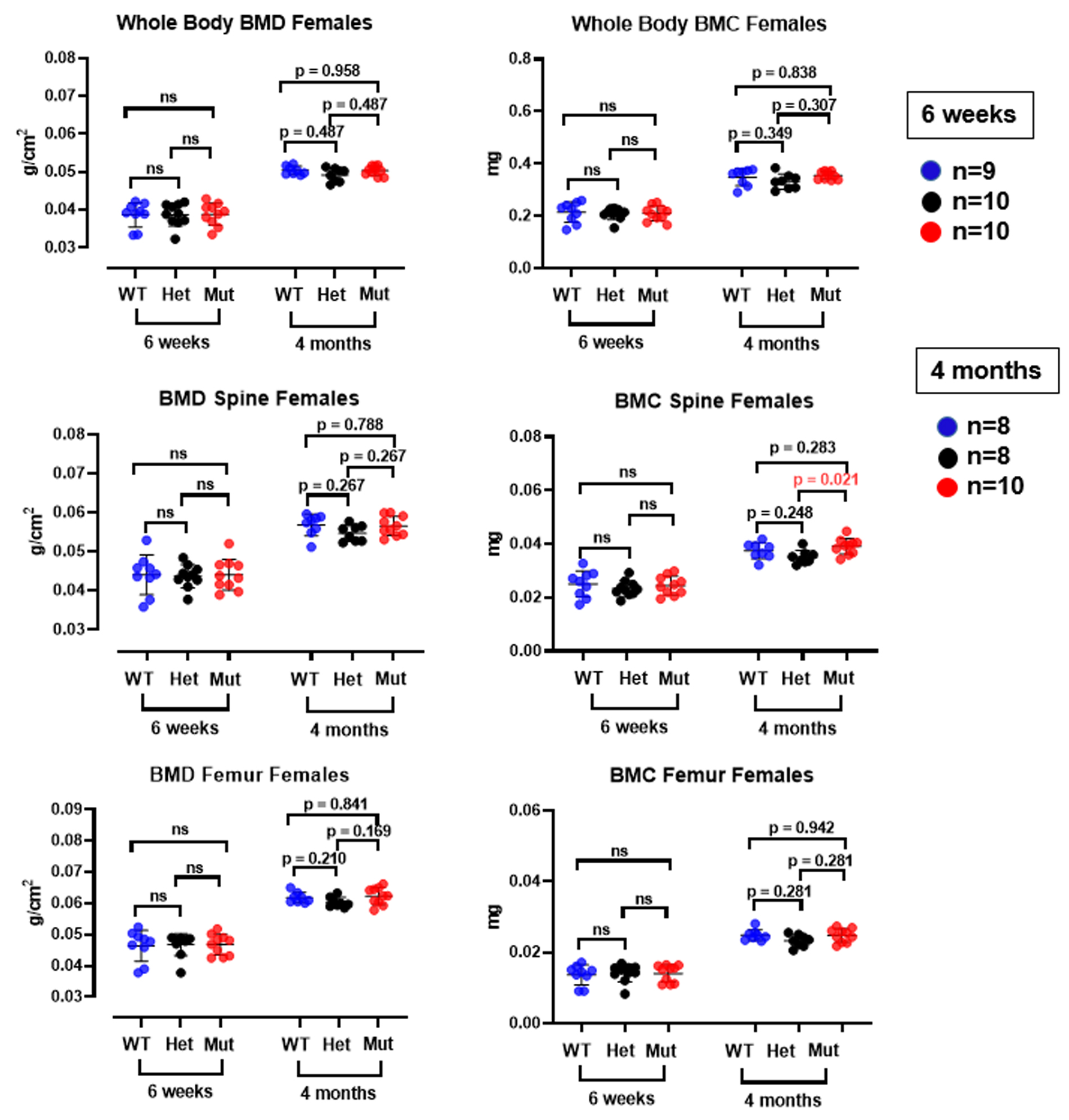

Supplement: Supplementary file 5 [file Image2.JPEG]

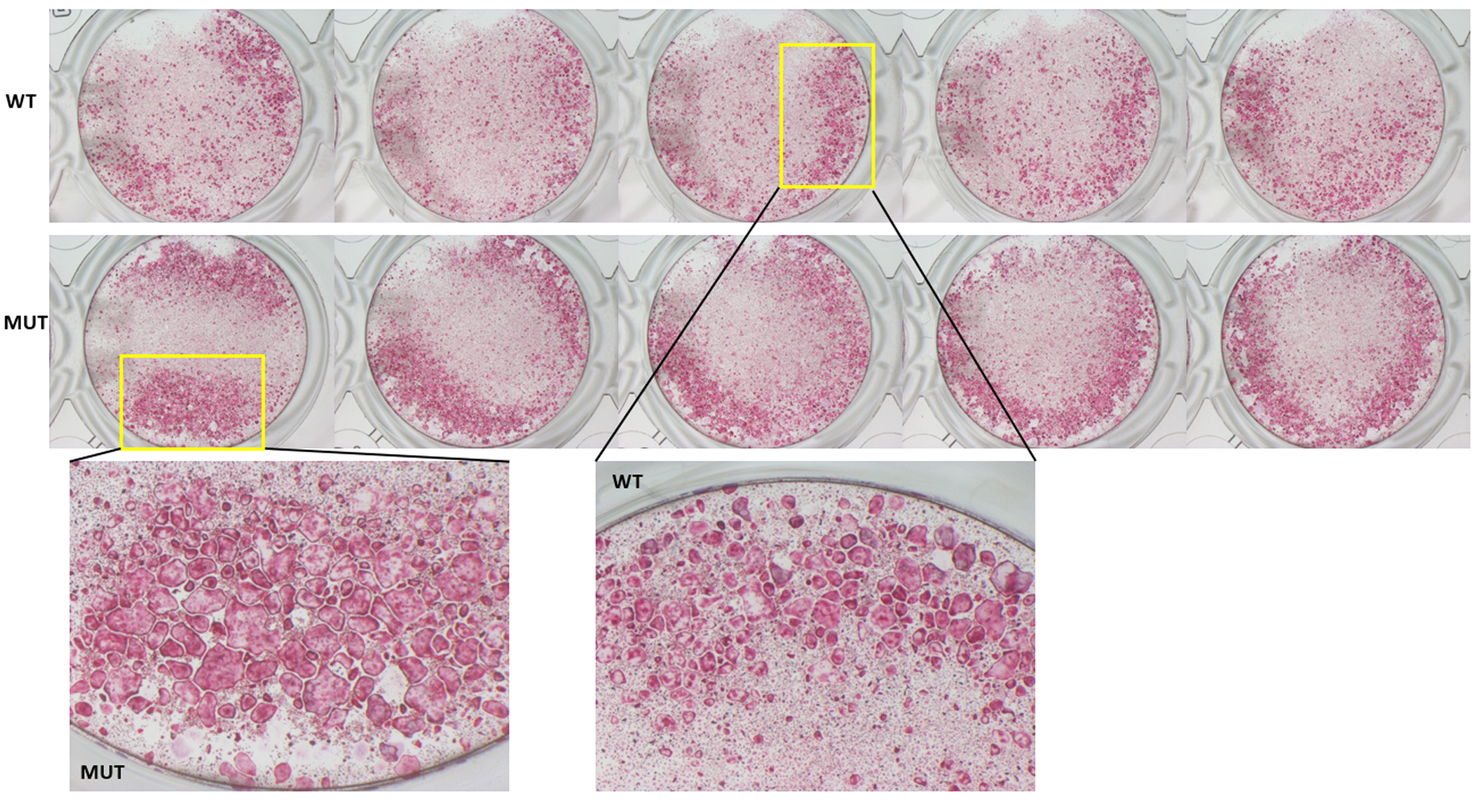

Supplement: Supplementary file 6 [file Image5.JPEG]
