# Supplementary material for: A Rab33b missense mouse model for Smith-McCort dysplasia shows bone resorption defects and altered protein glycosylation
Source: Front Genet. 2023 Jun 8;14:1204296. doi: 10.3389/fgene.2023.1204296 (PMC10285484; doi:10.3389/fgene.2023.1204296)
Supplement: Supplementary file 2 [file Table1.DOCX]

**SUPPLEMENTARY METHODS**

Mouse generation, Genotyping and Ethic Statement

For PCR genotyping, three primer pairs were used as follows: Rab33 (outer fwd) 5’- CGT CGT TTG TCA CTG CTA GG - 3’ and Rab33 (outer rev) 5’- GGC ACT CCG CTG AGA CAG - 3’, generating a 381 bp amplicon in all genotypes. Rab33 (Inner-For1): 5’– TCA TAG TGA TCG GTG ACA GCA ATG TGG GCC - 3’and Rab33 (outer rev) 5’- GGC ACT CCG CTG AGA CAG – 3, generating a 185 bp amplicon from the mutant allele. Rab33 (Inner rev): 5’-AGG CAC GTC TTG CCC ACG TTC GAG TCG - 3’ and Rab33 (outer fwd) 5’- CGT CGT TTG TCA CTG CTA GG - 3’, generating a 234 bp amplicon from the WT allele. The outer fwd and the outer rev primers need to be at 10uM final concentration, while the Inner For1 and the Inner rev need to be at 40uM final concentration. The PCR program was: 94°C for 2 min, then 32 cycles of 94°C for 30 s, 62°C for 30 s, and 72°C for 30 s, and lastly, 72°C for 5 min.

Cell culture and RNA Interference

Bone marrow-derived macrophages were obtained from femurs and tibias by cutting the epiphyses of the bones and eluting the bone marrow by centrifugation of the bones at 3000 rpm for 30 sec. The bone marrow was then resuspended in complete medium α-MEM + glutamine (Gibco Cat # 12561-056) + 15% FBS (Fetal Bovine Serum cat #S11550 R&D System), 100 units/mL penicillin, and 100 μg/mL streptomycin,and anti-mycotic (Anti-Anti 100X Gibco cat #15240-062) filtered with a 70-μm mesh cell strainer, centrifuged at 0.6g for 5 min. Red blood cells were removed with a commercially available solution 1X RBC Lysis Buffer Solution (eBioscience (cat #00-4333-57). Cell were then plated onto a cell culture dish with complete α-MEM 15%FBS plus 5% M-CSF overnight. Next day the non-adherent cells were collected and plated in Petri dish cells, expanded for 2-3 days with M-CSF. Cells were then harvested, counted, and seeded on cell culture plate (48 well plate) at 5X10^4^ with α-MEM 15% FBS complete, with addition of M-CSF (5%) and RANKL (100ng/ml). The medium was changed every 3 days. On day 4 the cells in the 48 well plate were fixed with 10% formalin and maintained at 4C^0^ until tartrate-resistance acid phosphatase (Trap) staining, which was performed according to standard procedures.

Both M-CSF and RANKL used in the osteoclast assay were kindly provided by Dr. Bryant G Darnay (MD Anderson Cancer Center) as conditioned medium from L929 cells (used at 5%) and as His-RANKL purified recombinant protein, respectively.

Western blot, immunofluorescence, and lectin staining

To prepare mouse liver samples for WB, isolated liver was washed 3x with PBS, cut with razor blade, and homogenized in 2 ml Potter homogenizer with 20 strokes in 2 ml of PBS with protease inhibitors/1 mg of tissue). Small aliquot of the lysate was examined under the microscope to confirm the absence of unbroken cells. Resulting slurry was centrifuged at 1000xg for 5 min to remove unbroken material. 50 μl of each variant was taken and saved as total lysate sample before centrifugation. Supernatant was transferred to the Beckman 1.5 ml tube and centrifuged at 120000xg for 1 hr in Max XP Ultracentrifuge using TLA55 rotor. 50 μl of each variant supernatant was collected as cytosol sample. Membrane pellet was resuspended in 0.5 ml of hot 2%SDS/1 mg of tissue as membrane-enriched sample. Samples were sonicated, mixed with 6x loading buffer containing β-mercaptoethanol and heated for 10 min at 70°C. Membrane-enriched and total lysate samples (10 μl each) were loaded into Genescript (8–16%) gradient gel for Coomassie staining to ensure that protein concentration is similar for different samples.

Micro-Computed Tomography

The distal femurs, from the midshaft down to the epiphysis, and L3 vertebras were scanned with an energy of 55 kVp, X-ray tube current of 114 mA, and 200 ms integration time. For the quantification of trabecular bone of the distal femur metaphysis, the region of interest was comprised of 151 contoured transverse slices extending 1.8 mm above the distal growth plate, applying a grayscale threshold (lower threshold 220, upper threshold 1000) and Gaussian noise filter (sigma 0.8, support 1). The quantification of the cortical thickness was performed in 20 contoured transverse slices at the femoral midshaft region, using the previous settings but with a lower threshold of 260. For the trabecular bone quantification of the L3 vertebrae, the region of interest for analysis comprised the entire vertebral body, including the maximum number of slices possible between both growth plates. Fractional bone volume (bone volume/tissue volume; BV/TV) and architectural properties of trabecular bone such as trabecular thickness (Tb.Th, μm), trabecular number (Tb.N, mm−1), and connectivity density (Conn. D, mm−3) were calculated using previously published methods (Suva et al., 2008). Femoral cortical geometry was assessed in a region centered at the femoral midshaft. The outer surface of the bone was found automatically using the manufacturer's built-in contouring tool. Total area was calculated by counting all voxels within the contour and bone area by counting all voxels that were segmented as bone. Marrow area was calculated as total area minus bone area. This calculation was performed on all 20 slices (1 slice = 12.5 μm), using the average for the final calculation. The outer and inner perimeter of the cortical midshaft was determined by a three-dimensional triangulation of the bone surface (BS); other cortical parameters were determined as described (Suva et al., 2008).
